# Integrating Analysis to Identify Differential circRNAs Involved in Goat Endometrial Receptivity

**DOI:** 10.3390/ijms24021531

**Published:** 2023-01-12

**Authors:** Wenjing Wang, Xupeng Zang, Yaokun Li, Dewu Liu, Linjun Hong, Guangbin Liu

**Affiliations:** 1 College of Animal Science, South China Agricultural University, Guangzhou 510642, China; 2 National Local Joint Engineering Research Center of Livestock and Poultry, South China Agricultural University, Guangzhou 510642, China

**Keywords:** endometrium receptivity, goat, circRNAs, maternal recognition of pregnancy, ISG15

## Abstract

Endometrial receptivity is one of the main factors underlying a successful pregnancy, with reports substantiating the fact that suboptimal endometrial receptivity accounts for two-thirds of early implantation event failures. The association between circRNAs and endometrial receptivity in the goat remains unclear. This study aims to identify potential circRNAs and regulatory mechanisms related to goat endometrial receptivity. Therefore, the endometrial samples on day 16 of pregnancy and day 16 of the estrous cycle were analyzed using high-throughput RNA-seq and bioinformatics. The results show that 4666 circRNAs were identified, including 7 downregulated and 11 upregulated differentially expressed circRNAs (DE-circRNAs). Back-splicing and RNase R resistance verified the identified circRNAs. We predicted the competing endogenous RNA (ceRNA) regulatory mechanism and potential target genes of DE-circRNAs. Gene Ontology (GO) and Kyoto Encyclopedia of Genes and Genomes (KEGG) analyses of these predicted target genes suggest that DE-circRNAs were significantly involved in establishing endometrial receptivity. Furthermore, Sanger sequencing, qPCR, correlation analysis and Fluorescence in Situ Hybridization (FISH) show that circ_MYRF derived from the host gene myelin regulatory factor (*MYRF*) might regulate the expression of interferon stimulating gene 15 (*ISG15*), thereby promoting the formation of endometrial receptivity. These novel findings may contribute to a better understanding of the molecular mechanisms regulating endometrial receptivity and promoting the maternal recognition of pregnancy (MRP).

## 1. Introduction

Successful embryo implantation is essential for normal pregnancy development in all mammals, while a receptive endometrium is a crucial prerequisite for embryo implantation [1]. Studies have shown that the acquisition of endometrial receptivity is a spatiotemporal process, and a large amount of crosstalk occurs between the endometrium and conceptus, which is also known as the “window of implantation” [2]. During this period, the proliferation of endometrial stromal cells and the differentiation of epithelial cells change the morphology and structure of the endometrium, resulting in the endometrium having a receptive capacity, thereby completing embryo implantation [3,4]. Previous studies have shown that endometrial receptivity is regulated by ovarian hormones, growth and transcription factors, lipid mediators and cytokines with paracrine signaling [5,6]. The dysfunctional receptive endometrium could cause infertility [7]. In ruminants, the establishment of endometrial receptivity accompanies the maternal recognition of pregnancy (MRP). MRP was successfully established due to the effect that embryonic-derived interferon τ (IFNτ) plays in corpus luteum roles by inhibiting the pulsatile release of prostaglandin F2α (PGF2α) in the goat endometrium [8,9]. During this period, and stimulated by these hormones and IFNτ, the endometrial epithelium undergoes dynamic changes to become receptive, which is critical for anchoring the implanting embryo to the apical surface of the luminal epithelium [10]. Presently, ample evidence suggests that several other molecules regulate endometrial receptivity [11,12]. Remarkably, a dysfunctional receptive endometrium can cause infertility [7]. Current evidence suggests that 30% of implantation failures may be attributed to embryo quality, whereas the remaining 70% result from poor uterine receptivity [13,14]. Accordingly, it is necessary to thoroughly investigate the molecular mechanisms regulating endometrial receptivity.

As a large class of non-coding RNAs, circular RNAs (circRNAs) are produced by the back-splicing of precursor mRNAs and are characterized by the 3′ and 5′ ends covalently linked to form a covalently closed loop [15,16]. It is well-recognized that circRNAs, with a unique circular structure, are more stable and have longer half-lives than mRNAs [17]. Notably, previous studies found that most circRNAs composed of one or more exons are conserved among different species but exhibit temporal and spatial specificity in different tissues and developmental stages of the same species [18]. Although the functions of most circRNAs remain unclear, previous studies have shown that circRNAs have molecular functions of regulating gene expression. The competing endogenous RNA (ceRNA) hypothesis states that mRNAs, lncRNAs, circRNAs and transcribed pseudogenes can communicate with and regulate each other through miRNA response elements (MREs) [19]. Previous studies have shown that ciRS-7 is one of the highly expressed circRNAs in the brains of humans and mice, and acts as microRNA sponges to bind miR-7 in nerve tissue to hinder midbrain development [20,21]. Some circRNAs involved in endometrial receptivity were identified in goats [4]. For instance, circRNA-9119 can reportedly regulate the receptive endometrium development of dairy goats through a circRNA-9119-miR-26a-PTGS2 pathway [22]. In contrast, circRNA8073 is regarded as a miRNA sponge of miR-181a that can reduce its expression level, thereby indirectly increasing the abundance of neurotensin in the endometrium and promoting the establishment of endometrial receptivity [23]. The overall analysis on the regulation of endometrial receptivity by circRNAs in endometrium, nevertheless, is still lacking.

In this study, we performed RNA sequencing of the circRNAs present in goat endometrial samples on day 16 of pregnancy (P16) and nonpregnant goats on day 16 of the estrous cycle (C16). Subsequently, qRT-PCR combined with ceRNA interaction network construction were performed to identify potential circRNAs in the endometrium linked to endometrial receptivity. Our findings provide novel insights indicating that circMYRF is associated with the regulation of *ISG15* expression during the window of MRP in the doe, which may provide the foothold for improving the efficiency of RMP.

## 2. Results

### 2.1. Identification and Characterization of circRNAs in the Goat Endometrium

**Figure 1:**
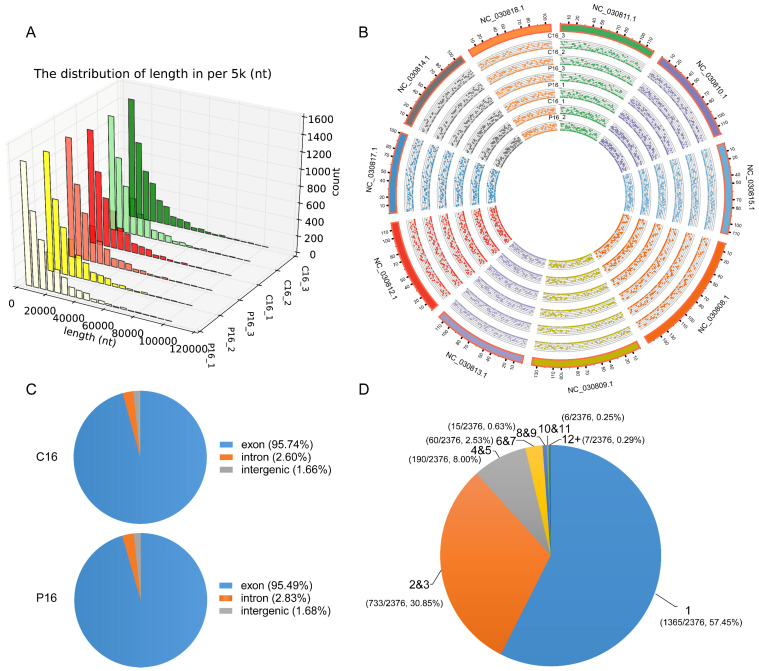
Identification of circRNAs. (**A**) The full-length distribution of circRNAs of all goat endometrium tissue samples. Each column represents 5000 nt. The *x*-axis length (nt) represents the length distribution of full-length circRNA; the *y*-axis represents different samples; the *z*-axis (count) represents the number of circRNAs. (**B**) The Circos plot shows the distribution of circRNAs on goat chromosomes. From the outside to the inside, the outside layer indicates the top 10 chromosome map of the goat genome, and the inside layers denote the distribution of circRNAs of each sample on these chromosomes. From outside to inside, the samples are C16_3, C16_2, P16_3, P16_1, C16_1 and P16_2, respectively. (**C**) The pie charts show the genic distribution of circRNAs in P16 and C16, respectively. (**D**) The amount of circRNAs produced by the host gene. Different colors represent different numbers of circRNAs produced by host genes. The values in parentheses represent the number and proportion of host genes that produce a corresponding number of circRNAs in total host genes.

The Illumina paired-end RNA-seq approach was used to purify and sequence RNAs for identifying circRNAs and their corresponding changes in expression levels between the P16 and C16 goat endometrium. A total of over 400 million raw reads were obtained from the endometrium for these two stages, and the quality control results of the data are shown in Table S1. We obtained a total of 4666 circRNAs from these data, and the full-length distribution was mainly concentrated below 5000 nt (Figure 1A). The density of identified circRNAs among different chromosomes was not uniform (Figure 1B). After comparing with the database, we observed that the 95.74% and 95.49% of circRNAs from C16 and P16, respectively, were extensively transcribed from the exon region, and the remaining fraction were derived from the intron and intergenic region (Figure 1C). Following this, further analysis shows that most host genes could produce only one circRNA, although many genes still produced multiple circRNAs. In addition, more than 11% of host genes generated more than 3 circRNAs per gene, and even 7 host genes produced more than 12 circRNAs (Figure 1D).

**Figure 2:**
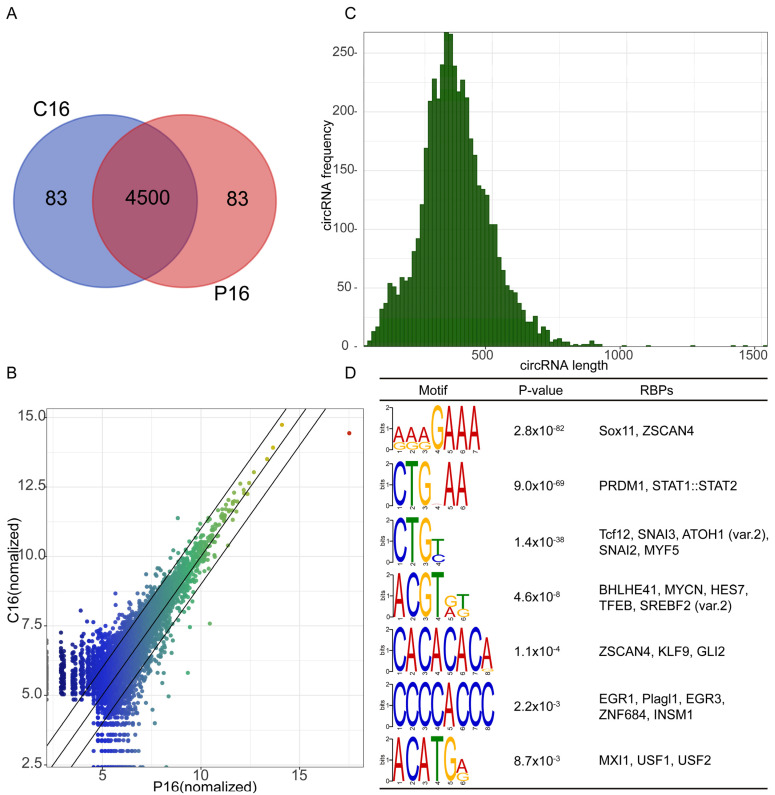
Characterization of circRNAs. (**A**) Venn diagram showing circRNAs co-expressed and specifically expressed in the goat endometrium of P16 and C16. (**B**) The overall analysis of the circRNA expression levels between the P16 and C16 endometrium. (**C**) The splice-length distribution of circRNAs. (**D**) The RRMs of RBPs enriched in the flanking regions of the circRNA junction sites.

As a result, among the 4666 circRNAs obtained in the two stages, 4500 circRNAs were co-expressed in these two stages, and 83 circRNAs were specifically expressed in each stage of P16 and C16 (Figure 2A,B). To study the molecular characteristics of circRNAs, we further analyzed the length of mature circRNAs after splicing, which primarily ranged from 200 to 500 bp (Figure 2C). Furthermore, it is widely acknowledged that RNA binding proteins (RBPs) play a major role in RNA metabolism, including regulating RNA splicing, maturation and function [24] and RBPs usually contain at least one RNA recognition motif (RRM) [25]. We hypothesized that RBPs in the flanking regions of the circRNA junction sites might potentially regulate circRNA biogenesis in different physiological processes; therefore, we analyzed the potential RBPs of these identified circRNAs. Notably, we identified some RBPs, including EGR1, EGR3, ZNF684, INSM1, ZSCAN4, KLF9 and GLI2, whose binding motifs were enriched in the flanking regions of circRNA junction sites, implying that these RBPs may play functional roles in circRNA biogenesis (Figure 2D).

### 2.2. GO and KEGG Analysis of Host Genes of circRNAs

**Figure 3:**
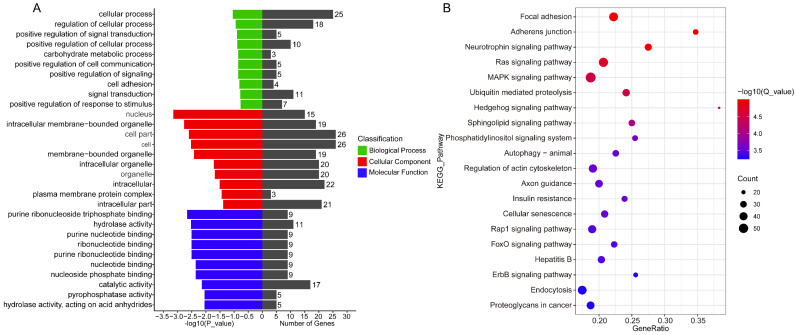
The biological function analysis of the host gene of circRNAs. (**A**) The GO enrichment analysis of host genes of circRNAs. The right *x*-axis indicates the number of gene in a category, and the left *y*-axis indicates the specific category of GO. Green: biological process, red: cellular component and blue: molecular function. (**B**) Scatter plot shows the KEGG pathway enrichment analysis of the host gene of circRNAs.

Previous studies have shown that circRNAs could exert biological functions by regulating the expression of their host genes. Therefore, we performed GO enrichment and KEGG pathway analyses on the host genes of circRNAs to explore their potential physiological functions. Five of the top 10 GO terms in biological processes were involved in the processes of cellular changes, including cellular processes (GO:0009987), regulation of cellular processes (GO:0050794), positive regulation of cellular processes (GO:0048522), positive regulation of cell communication (GO:0010647) and cell adhesion (GO:0007155) (Figure 3A and Table S2). Furthermore, the KEGG pathway analysis yielded 296 enriched signaling pathways (Table S3). Among the top 20 signaling pathways, focal adhesion (chx04510), the MAPK signaling pathway (chx04010), the Ras signaling pathway (chx04014) and Adherens junction (chx04520) were associated with endometrium development (Figure 3B).

### 2.3. Identification of DE-circRNAs in the P16 and C16 Endometrium

**Figure 4:**
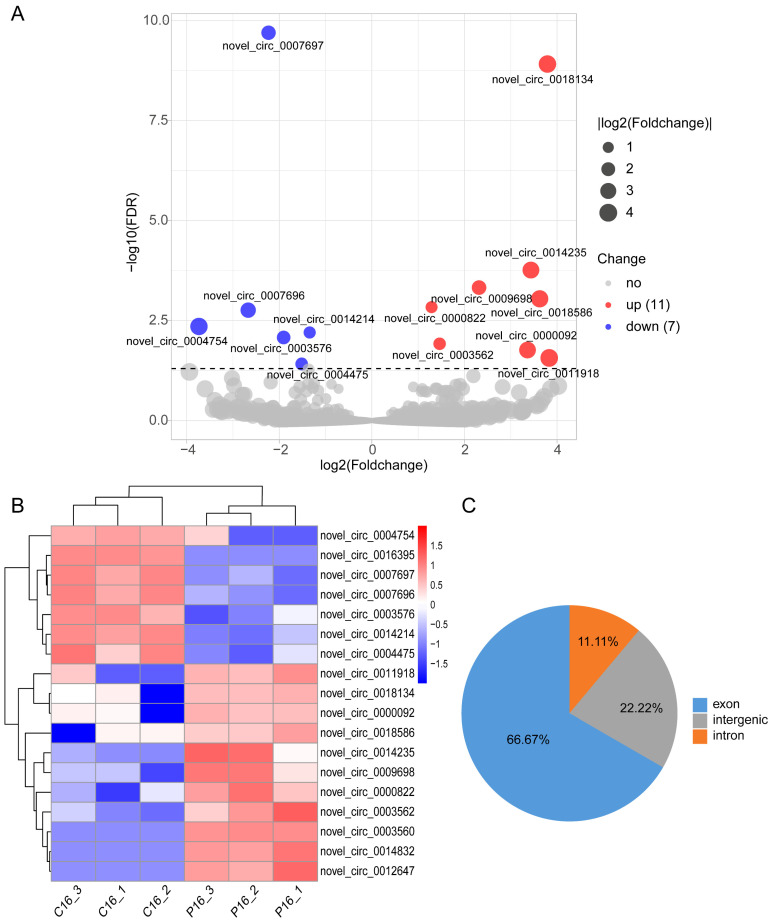
The detected DE-circRNAs in goat endometrium. (**A**) Volcano plot visualization of the statistical difference of the DE-circRNAs. The horizontal axis represents the fold-change of detected circRNAs, and the vertical axis represents the FDR. Red, up-regulated circRNAs; blue, down-regulated circRNAs; gray, not significantly changed circRNAs. (**B**) Hierarchical clustering shows the expression profiles of all DE-circRNAs. Each row represents one DE-circRNA, while columns represent different samples. The color scale is from −2.0 (blue, lower circRNA expression level) to 2.0 (red, higher circRNA expression level). (**C**) The pie chart shows the genic distribution of DE-circRNAs.

To reveal DE-circRNAs in the goat endometrium during the two development stages, we focused on circRNAs. A total of 18 DE-circRNAs (11 upregulated and 7 downregulated) were obtained in the P16 and C16 endometrium using the criteria FDR < 0.05 and |log2(fold change)| > 1 (Figure 4A and Table S4). Hierarchical clustering analysis shows a clear distinction of DE-circRNAs between the P16 and C16 endometrial samples (Figure 4B). Furthermore, 66.67% of DE-circRNAs were extensively spliced from exon regions, and the proportion of intergenic circRNAs in identified DE-circRNAs was higher than in the full list of circRNAs identified in goat endometrium (Figure 1C and Figure 4C). Interestingly, the novel_circ_0003560 and novel_circ_0003562 in these DE-circRNAs were derived from one host gene, circ_LOC106502060 (Table S4).

### 2.4. Prediction and Construction of ceRNA Regulatory Network

**Figure 5:**
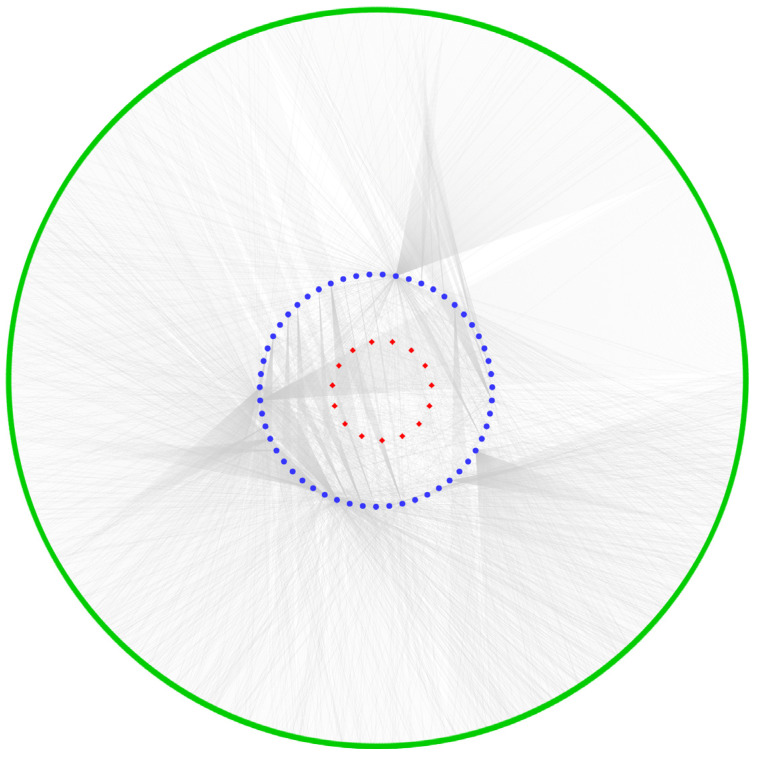
The ceRNA interaction network. The innermost red nodes represent DE-circRNAs. The blue nodes in the middle circle represent predicted miRNA targets. The green outer circle represents the predicted mRNAs.

Previous studies have confirmed that the circRNAs function as miRNA sponges competitively bind miRNAs, and indirectly regulate miRNA-mediated target gene expression [26,27]. To explore the functional mechanism of circRNAs involved in endometrial receptivity, we predicted the potential circRNAs-miRNAs interactions for DE-circRNAs. The result indicates that a total of 55 target miRNAs identified to the 15 DE-circRNAs while 1968 mRNAs were bound by the 55 miRNAs and suggests that a single circRNA regulates multiple miRNAs and mRNAs (Figure 5).

### 2.5. Functional Annotation of DE-circRNAs

**Figure 6:**
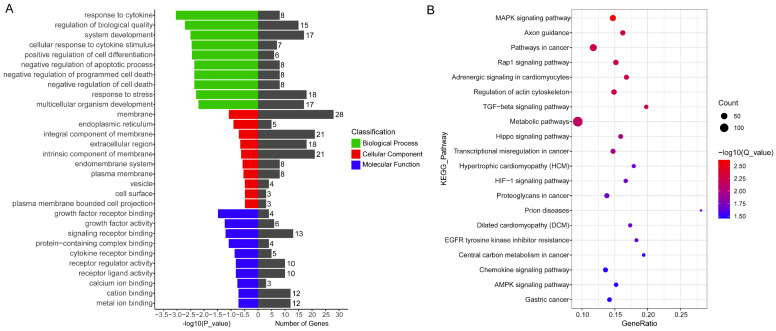
Functional analysis of the ceRNA network. (**A**) GO enrichment analysis of the ceRNA network. (**B**) KEGG pathways analysis of the ceRNA network.

GO enrichment and KEGG pathway analyses were performed on the predicted target genes of DE-circRNAs to analyze the potential functions of DE-circRNAs in endometrial receptivity. During GO enrichment analysis, 612 GO terms were enriched, including a response to cytokine (GO:0034097), a cellular response to cytokine stimulus (GO:0071345), positive regulation of cell differentiation (GO:0045597), negative regulation of cell death (GO:0060548) and negative regulation of programmed cell death (GO:0043069) (Figure 6A and Table S5). Meanwhile, KEGG pathway analysis shows that DE-circRNAs were involved in regulating 316 signaling pathways, such as the MAPK signaling pathway (chx04010), TGF-beta signaling pathway (chx04350), Rap1 signaling pathway (chx04015), regulation of actin cytoskeleton (chx04810) and metabolic pathways (chx01100) (Figure 6B and Table S6), which significantly influenced the cellular processes involved in goat endometrium development.

### 2.6. Validation of DE-circRNAs in the Goat Endometrium

**Figure 7:**
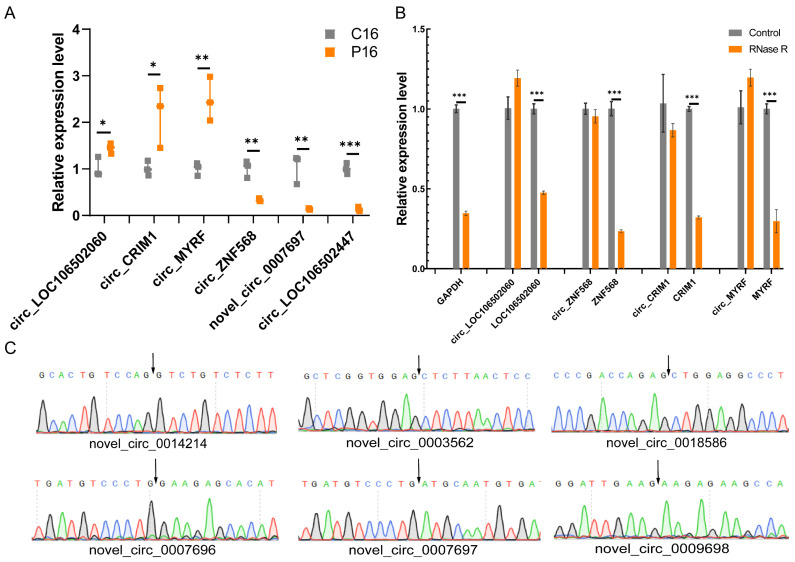
Validation of DE-circRNAs in the goat endometrium. (**A**) The relative expression level of DE-circRNAs were measured by qPCR in P16 and C16. (**B**) Validation of the resistance of DE- circRNAs and mRNAs to RNase R. There are three independent replicates per group, and the data are shown as the mean ± standard error of the mean (SEM) values. *, *p* < 0.05; **, *p* < 0.01; ***, *p* < 0.001. (**C**) The head-to-tail splice junctions for circRNAs were confirmed by Sanger sequencing. Black arrows represent the junction sites.

Six DE-circRNAs were randomly selected to design primers (Table S7) in their junction sites and validate the identified circRNAs from the RNA-seq data. The qPCR results revealed that the expression levels of novel_circ_0007697, circ_LOC106502447 and circ_ZNF568 were significantly lower, while circ_CRIM1, circ_MYRF and circ_LOC106502060 levels were significantly higher in P16 compared to C16, which is consistent with the RNA sequencing data (Figure 7A). In addition, the RNA samples of C16 and P16 were treated with exonuclease RNase R to verify the resistance. The results show that the expression levels of these circRNAs after RNase R treatment were not significantly different from controls, while the linear gene expressions were significantly decreased (Figure 7B). Meanwhile, Sanger sequencing confirmed the presence of head-to-tail splice junctions in the circRNAs (Figure 7C). These results further suggest that circRNAs have covalently closed circular structures and indicate that the RNA-seq data are reliable.

### 2.7. Functional Prediction of circ_MYRF in Goat Receptive Endometrium

**Figure 8:**
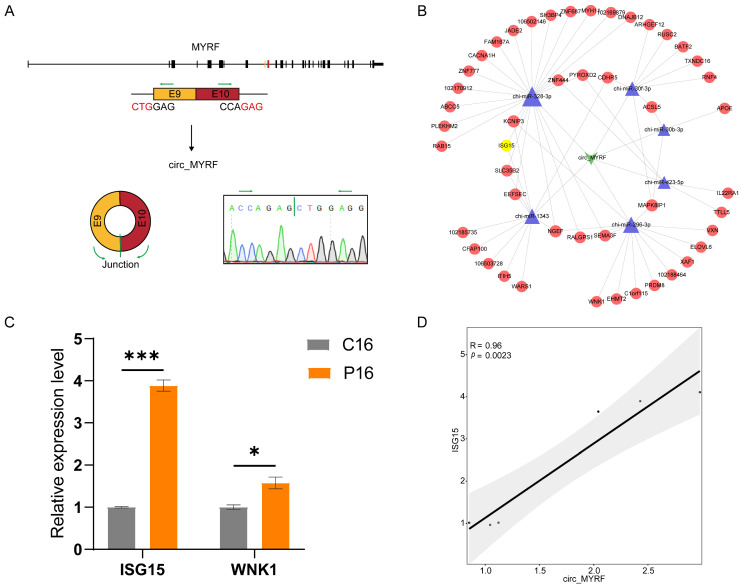
The characterization and putative function of circ_MYRF in the goat endometrium. (**A**) The genomic location of circ_MYRF in the host gene, *MYRF*. The expression of circ_MYRF was validated by qPCR and Sanger sequencing. Arrows represent divergent primer binding sites in the circular junction of circ_MYRF. (**B**) A putative ceRNA network of circ_MYRF. The green quadrilateral represents circ_MYRF, the blue triangles represent targeted miRNAs, and the red circles denote targeted mRNAs. The yellow circle represents the *ISG15* mRNA. (**C**) Relative expression level of two randomly selected target genes in the ceRNA network determined by qPCR. Values represent mean ± SEM. *, *p* < 0.05; ***, *p* < 0.001. (**D**) The correlation between circ_MYRF expression and *ISG15* expression.

**Figure 9:**
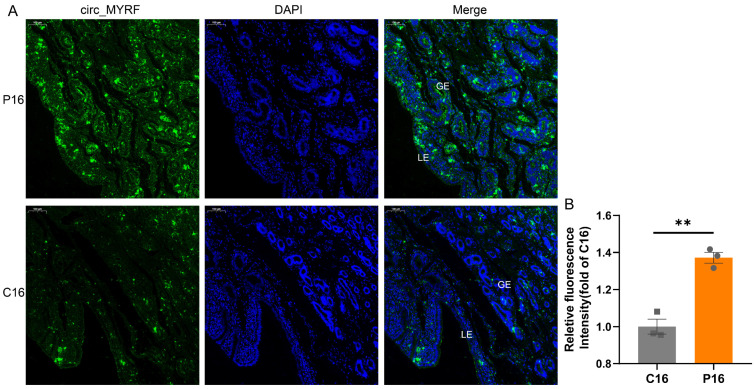
FISH analysis of circ_MYRF in the C16 and P16 uterus. (**A**) Representative images of circ_MYRF staining for two stages. The circ_MYRF was abundantly expressed in GE and stroma of P16, while it was slightly expressed in C16. LE, endometrial luminal epithelium; GE, glandular epithelium. Scale bar: 100 µm. (**B**) Quantitative analysis was performed by measuring the fluorescence intensity of circ_MYRF in two stages. The data were shown as the mean ± SEM. **, *p* < 0.01.

During the screening of candidate circRNAs, circ_MYRF (log2(fold change) = 3.63; FDR = 0.0009, exonic circRNA) attracted our interest. We found that circ_MYRF was derived from 2 exons, including exons 9 (40573323-40573399) and 10 (40573581-40573691) of the host gene *MYRF* (NC_030836.1) (Figure 8A). We constructed one ceRNA network showing the relationship among circ_MYRF-miRNAs-mRNAs, including six putative miRNA sponges and 56 targeted genes (Figure 8B). Subsequently, we used qPCR to examine the expression levels of two randomly selected target genes, and the results were in line with the expression of circ_MYRF (Figure 8C). Interestingly, interferon stimulating gene 15 (*ISG15*) was one of the target genes of circ_MYRF in the network analysis. ISG15 is well-established as one of several proteins generated by conceptus-derived Type I and/or a Type II interferon and can reportedly regulate endometrial receptivity and conceptus development [28]. Correlation analysis confirmed that the expression of circ_MYRF was significantly associated with the *ISG15* mRNA expression (Figure 8D). FISH analysis was performed to determine the circ_MYRF location in the endometrium tissues of P16 and C16 (Figure 9). It is abundantly expressed in the uterine glandular epithelium (GE) and stroma, which is consistent with *ISG15* expression in the endometrial tissue, as reported in a previous study [29].

## 3. Discussion

Current evidence suggests that endometrium receptivity determines successful embryo implantation and embryonic mortality [30]. It is essential to conduct a comprehensive study of the molecular regulation underlying endometrial receptivity of specificity to pregnancy in the doe. The past decade witnessed the advent of high-throughput RNA sequencing, enabling us to better understand the molecular regulation processes of endometrial receptivity. In a previous study, it was revealed that hundreds of genes are involved in regulating endometrial receptivity by transcriptome studies [31]. In addition, it has reported that dozens of miRNAs found in both humans and mice were could potentially modulate endometrial receptivity [3]. In this study, we investigated the circRNA profiles of goat endometrium at C16 and P16 and identified circRNAs involved in regulating endometrial receptivity. Importantly, our research expanded the repertoire of goat endometrium-expressed circRNAs and provided information for future studies on endometrial development and embryo implantation. Notably, the RNAs used in this study were not treated with RNase R to remove all linear RNAs; however, they were predicted by the unique back-splicing structure of circRNAs, which may have some limitations in parsing all circRNA expression profiles.

A previous study has shown that circRNAs are formed by back-splicing the corresponding linear transcript [32]. In the present study, we found that novel_circ_0003560 and novel_circ_0003562 were derived from one host gene, indicating that diverse circRNAs could be expressed by a single gene locus, which is consistent with previous results [33]. Furthermore, growing evidence suggests that circRNAs could regulate the expression of their host gene to participate in the regulation of biological processes [32,34]. In this study, the host gene of the DE-circRNA, novel_circ_0009698 (circ_CRIM1), *CRIM1*, can be promoted by hormones and IFNτ in goat endometrium, and a deficiency of CRIM1 hindered cell proliferation, adhesion and prostaglandin secretion and thus disrupted normal endometrial receptivity [12,35,36], suggesting that it plays a vital role in the establishment of pregnancy. In contrast, circ_CRIM1 may contribute to cell proliferation, cell adhesion and the formation of normal endometrial receptivity by promoting CRIM1 expression. This hypothesis, however, should warrant validation in further study. In the study by Song et al. [4], there were 334 DE-circRNAs identified in the endometrium from goats at gestational day 5 and goats at gestational day 15 using Illumina Solexa technology. Interestingly, both Song’s study and ours found that the circRNA deriving from *CRIM1* gene was highly expressed in the receptive endometrium, which further suggests that circ_CRIM1 may be involved in regulating endometrial receptivity. In our study, most of the circRNAs were termed as exonic circRNAs. Intriguingly, we found that the proportion of intergenic circRNAs in identified DE-circRNAs was higher than in the full list of circRNAs identified in the goat endometrium. Substantial evidence suggests that exonic circRNAs can usually interact with host genes and regulate the roles of host genes in biological processes [37,38,39]. Since intergenic circRNAs do not have corresponding host genes, they might exert dominant functions by acting as miRNA regulators [40], suggesting that DE-circRNAs identified in this study may serve as miRNAs sponges to regulate the endometrial receptivity.

According to the ceRNA hypothesis, circRNAs are molecular sponges of miRNAs that ultimately regulate mRNA expression [19]. For instance, the ciR3175-miR182-TES pathway was identified in the endometrium of dairy goats; ciR3175 regulates the expression of TES by adsorbing miR182 and then decreases the expression of BCL-2/BAX through the MAPK pathway, thereby inhibiting EEC apoptosis [41]. This regulatory mechanism of circRNAs indicates that there are communication networks among RNAs. In our study, a circRNAs-miRNA-mRNA network analysis performed on DE-circRNAs shows that most DE-circRNAs predicted only one or two target sites for miRNAs, which is consistent with the literature [42]. This result suggests that circRNAs can act as a miRNA sponge and do not require many target sites.

It was reported that the receptive endometrium results from the normal development of the endometrium following successful pregnancy recognition. In addition to the dominant pregnancy recognition hormones, including PGF2α and IFNτ, many growth factors, cytokines and inflammatory factors can coordinate the hormones mentioned above to co-regulate the process [43,44]. Notably, circ_0012647, which is specifically expressed in P16 endometrium, was predicted to upregulate OAS1 expression by sponging miR-671-5p. Previous studies confirmed that OAS1 expression in luteal cells could be increased under the function of IFNτ to maintain corresponding corpus luteum roles [45,46], suggesting that circRNAs may cooperate with IFNτ to act on the corpus luteum to establish a successful pregnancy.

Subsequently, a functional analysis of the putative target genes was performed. During GO annotation, most target genes were enriched in cell-related biological processes, including a response to cytokine (GO:0034097), a positive regulation of cell differentiation (GO:0045597) and a negative regulation of the apoptotic process (GO:0043066). Mounting evidence substantiates that the endometrial events are mediated by cell proliferation, differentiation and apoptosis [47,48]. In the present study, the KEGG pathway analysis of target genes shows that pathways such as the MAPK signaling pathway (chx04010), the Rap1 signaling pathway (chx04015) and regulation of actin cytoskeleton (chx04810) were enriched. Previous studies have confirmed that the MAPK signaling pathway could be involved in regulating EEC proliferation [49], the Rap1 signaling pathway was involved in regulating the function of endometrial stromal cells [50] and the regulation of actin cytoskeleton participated in regulating the remodeling of adherens junctions [51]. Overall, these results indicate that circRNAs may regulate goat endometrial receptivity through these pathways by ceRNA competition regulation mechanisms.

We further explored the detailed regulatory mechanism of circRNAs. ISG15 is a ubiquitin homolog whose expression is induced by the conceptus IFN in a temporal and cell-specific manner in the uterus, to degrade proteins detrimental to fetal/embryo survival [52]. Previous literature suggests that ISG15 plays a critical role in determining endometrial receptivity and regulating embryo development [28,53]. Chandrakar et al. consistently found an increase of ISG15 mRNA concentration in the goat endometrium during the early stages of pregnancy (16–24d) [54]. In this study, ceRNA network analysis and qPCR analysis shows that, compared with C16, the mRNA expression level of ISG15 was higher in P16. Moreover, the FISH analysis results show that circ_MYRF was abundantly expressed in the GE and stroma of P16 and slightly expressed in C16. In contrast, the ISG15 mRNA was also mainly localized in GE and stromal cells and exhibited limited localization in the luminal epithelium (LE) [29]. Hence, we hypothesize that circ_MYRF may regulate goat endometrial receptivity by targeting ISG15 in GE and stroma. This also suggests that circ_MYRF may serve as a potential biomarker to identify non-receptive endometrium and a therapeutic target to improve human endometrial receptivity for the treatment of infertility. Future mechanistic studies should be warranted to confirm the precise roles of circ_MYRF and ISG15 in pregnancy establishment.

## 4. Materials and Methods

### 4.1. Animals and Sample Collection

This study complied with the Ethical Principles in Animal Research, and was performed in accordance with the ethical standards of the Animal Care and Use Committee of South China Agricultural University (permit number: SYXK-2022-0136). Six healthy and disease-free primiparous Chuanzhong black goats (Capra hircus) were provided by Guangdong Wen’s Foodstuffs Group Co., Ltd. (Yunfu, China), and were randomly divided into a cyclic group (*n* = 3) and a pregnancy group (*n* = 3). Goats that belonged to the pregnant group were twice artificially inseminated using extended semen from one ram at the onset of estrus (day 0) and 12 h after. Subsequently, the goats were slaughtered at the local slaughterhouse on day 16 of the estrus cycle (C16) or pregnancy (P16). For each animal, the uterus was quickly removed and transported to the laboratory in an icebox, and pregnancy was confirmed by the presence of apparently normal filamentous conceptuses during uterine flushing [55]. The uteri were opened longitudinally along the antimesometrial side. Approximatly 1 cm2 of endometrial tissue samples were taken from the middle of each uterine horn at the antimesometrial side of the uterus, and endometrial samples were snap-frozen in liquid nitrogen and stored at −80 °C for RNA extraction.

### 4.2. Library Preparation and RNA Sequencing

Total RNA was isolated from the endometrium using Trizol reagent (Invitrogen, Carlsbad, CA, USA) following the manufacturer’s procedure. Total RNA quality and concentration was checked using the NanoDrop 2000 equipment (Thermo Scientific, Waltham, MA, USA), and integrity was assessed using the RNA Nano 6000 Assay Kit of the Bioanalyzer 2100 system (Agilent Technologies, CA, USA). High throughput transcriptome sequencing was carried out by an Illumina Hiseq platform at Novogene (Beijing, China). Briefly, about 3 µg of total RNA per sample removed ribosomal RNAs using the Epicentre Ribo-zeroTM rRNA Removal Kit (Epicentre Madison, WI, USA), and rRNA free residue was cleaned up by ethanol precipitation. Then, the rRNA-depleted RNA was used to generate the sequencing library using the NEBNext^®^ UltraTM Directional RNA Library Prep Kit (NEB, Ipswich, MA, USA), according to the recommendations of the manufacturer.

### 4.3. RNA-seq Data Analysis

During this step, clean reads were obtained by removing adapter sequences, reads with more than 10% ploy-N and low-quality from the raw data. Simultaneously, Q20, Q30 and GC content of the clean reads were calculated, and all follow-up bioinformatics analyses were based on clean reads with high quality. Subsequently, the clean reads were mapped to the goat reference genome using Bowtie (v.0.12.9) [56]. Due to the high false-positive identification of circRNAs [57], we used two software, find_circ [21] and CIRI2 [58], to detect and identify circRNAs, and only circRNAs that were intersected between the two software were selected for further analyses.

### 4.4. Analysis of Differentially Expressed circRNAs (DE-circRNAs)

The expression level of circRNAs in each sample was counted and normalized with TPM [59]. The differential expression analysis between the P16 and C16 endometrium of goats was performed using the DESeq2 package [60]. The *p* values were adjusted using Benjamini-Hochberg’s approach for controlling the false discovery rate [61]. Differentially expressed circRNAs were identified using FDR < 0.05 and | log2(foldchange) | > 1 as screening criteria.

### 4.5. Motif Enrichment Analysis and ceRNA Network Construction

The 100 bp flanking region of the back-splicing site with circRNAs was retrieved from the goat genome, and then the short, ungapped motifs relatively enriched in these regions was compared with shuffled sequences and were detected using Dreme (v.5.1.1) [62]. The enriched motifs with *p* < 0.05 were selected for subsequent analysis. To associate the enriched motifs to potential RBPs, all selected motifs were compared against the JASPAR database [63] of known motifs using Tomtom (v.5.1.1) [64]. The top 5 target motifs with the most significant matches to the query motif were identified as potential RBPs, which might regulate the biogenesis of circRNAs.

The miRNA binding sites of the DE-circRNAs were predicted using the miRanda (v.3.3) software [65]. Then, miRNA-mRNA interactions were predicted using miRanda. Targetscan and RNAhybrid were used to determine the gene targets of each filtered miRNA. Using these data, the outline of the ceRNA regulatory network was generated using Cytoscape (v.3.7.2, http://www.cytoscape.org/, accessed on 30 October 2021) [66].

### 4.6. Functional Analysis of DE-circRNAs

To reveal the potential biological functions and principal pathways of DE-circRNAs of P16 and C16, Gene Ontology (GO) enrichment analysis and Kyoto Encyclopedia of Genes and Genomes (KEGG) analysis were used. The enriched GO terms of these target genes were identified using the clusterProfiler package in R, and the KEGG pathways were determined using KEGG Orthology Based Annotation System (KOBAS) [67].

### 4.7. Quantitative Real-Time PCR (qPCR)

Total RNAs were extracted from the same 6 samples of goat endometrium of P16 and C16 and used for RNA sequencing. Next, 2 µg of total RNA of each sample was incubated for 10 min at 37 °C with or without RNase R (3 U/µg RNA, GENESEED, Guangzhou, China), followed by inactivation for 10 min at 70 °C. All RNA samples were processed simultaneously to ensure equally effective RNase R treatment. The qPCR assay was conducted to quantify the amount of circRNA and mRNA. Initially, the first strand cDNA was synthesized using the Evo M-MLV RT Kit with gDNA Clean for qPCR II (Accurate Biology, Changsha, China). Then, qPCR reactions were conducted employing a SYBR^®^ Green Premix Pro Taq HS qPCR Kit (Accurate Biology, Changsha, China). The PCR volume was 10 μL, consisting of 1 μL cDNA, 0.2 μM of each primer, 5 μL 2 × SYBR Green Pro Taq HS Premix, 0.4 μM of ROX Reference Dye and RNase free water to make up the total volume. The thermal cycling conditions were as follows: 95 °C for 30 s followed by 40 cycles at 95 °C for 5 s and 60 °C for 30 s. Linear GAPDH was chosen as an internal reference to regulate the expression of circRNAs, and all reactions were performed in triplicate samples. To further identify the junction sequence of circRNAs, the RT-PCR products of divergent primers were analyzed by electrophoresis and Sanger sequenced at BGI Genomics Co., Ltd. (Shenzhen, China). The primer sequences used are listed in Table S7.

### 4.8. Fluorescence In Situ Hybridization (FISH) Analysis

The location of circ_MYRF in goat endometrium was determined by conducting a FISH analysis, as previously described [68]. In brief, micrometer sections (4 µm thick) were deparaffinized, digested with proteinase K and hybridized using FAM-labeled circ_MYRF probes (green). Simultaneously, cell nuclei were stained with DAPI. Images were then photographed using a positive fluorescence microscope (Nikon, Tokyo, Japan).

### 4.9. Statistical Analysis

Data were statistically analyzed using SPSS (version 26.0, SPSS Inc., Chicago, IL, USA), GraphPad Prism (version 8.0, Graphpad Software, San Diego, CA, USA) and R programming language (version 3.6). All qPCR results of circRNAs and mRNAs in goat endometrium of P16 were normalized using the calibrator group, C16, and the circRNA and linear mRNA treated with RNase R were normalized using control values. The Kolmogorov-Smirnov test was used to assess the normality of the data. All experiments were performed in three independent replicates, and data were expressed as the mean ± standard error of the mean (SEM). Differences between the two groups were analyzed using Student’s *t*-test. A *p* value < 0.05 was statistically significant, and *, **, and *** indicate *p* < 0.05, *p* < 0.01, and *p* < 0.001, respectively.

## 5. Conclusions

In summary, this study identified 4666 novel circRNAs, including 18 significantly differentially expressed circRNAs (11 upregulated and 7 downregulated). The ceRNA network and functional analyses of circRNAs suggests the potential roles of circRNAs in endometrial receptivity. Our study provides the circRNA expression profiles during early pregnancy and data on the estrus periods to study the molecular regulation mechanism of mammalian early pregnancy and further promotes research in embryo implantation, cancer and gynecological diseases.

## Data Availability

The datasets used and analyzed in this study can be found in online repositories. The names of the repository/repositories and accession number(s) can be found below: https://www.ncbi.nlm.nih.gov/, PRJNA578518.

## Supplementary Materials

The supporting information can be downloaded at: https://www.mdpi.com/article/10.3390/ijms24021531/s1.
