# Supplementary material for: Integrating Analysis to Identify Differential circRNAs Involved in Goat Endometrial Receptivity
Source: Int J Mol Sci. 2023 Jan 12;24(2):1531. doi: 10.3390/ijms24021531 (PMC9865150; doi:10.3390/ijms24021531)
Supplement: Supplementary file 1 [file ijms-24-01531-s001.zip › Supplementary Table S4.pdf]

Supplementary Table S4. DE-circRNAs in P16 and C16 endometrium

CircRNA ID	CircRNA Name	Up/Down	Log2(Fold Change)	FDR	Catalog	Start	End	Strand	Length	Chromosome
novel_circ_0004754	circ_ANO4	down	-3.73	0.0044652	exon	63488048	63549357	+	428	NC_030812.1
novel_circ_0007696	circ_LOC106502447	down	-2.67	0.0017456	exon	94379231	94394086	-	592	NC_030815.1
novel_circ_0007697	--	down	-2.23	2.01E-10	intergenic	94379231	94407339	-	873	NC_030815.1
novel_circ_0003576	--	down	-1.91	0.0084246	intergenic	25410497	25487125	+	666	NC_030811.1
novel_circ_0004475	circ_SLC38A1	down	-1.52	0.038394	exon	33471246	33488926	+	527	NC_030812.1
novel_circ_0014214	circ_ZNF568	down	-1.34	0.006314	exon	48046459	48062747	-	787	NC_030825.1
novel_circ_0016395	circ_OSBPL10	down	--	0.025534	exon	6249979	6304505	-	448	NC_030829.1
novel_circ_0000822	circ_SPICE1	up	1.29	0.0014668	intron	57957030	57965546	-	395	NC_030808.1
novel_circ_0003562	circ_LOC106502060	up	1.46	0.012089	exon	25358995	25410668	+	640	NC_030811.1
novel_circ_0009698	circ_CRIM1	up	2.32	0.00047589	exon	18763684	18765107	+	364	NC_030818.1
novel_circ_0000092	circ_RNF13	up	3.36	0.017341	exon	117834974	117898289	-	305	NC_030808.1
novel_circ_0014235	--	up	3.44	0.00017352	intergenic	50125988	50158133	+	292	NC_030825.1
novel_circ_0018586	circ_MYRF	up	3.63	0.00090739	exon	40573322	40573691	+	369	NC_030836.1
novel_circ_0018134	circ_ZCCHC24	up	3.79	1.22E-09	intron	10610220	10612264	+	258	NC_030835.1
novel_circ_0011918	circ_ZFPM2	up	3.83	0.027174	exon	22931496	22954428	+	261	NC_030821.1
novel_circ_0014832	--	up	--	4.52E-06	intergenic	5758305	5842135	+	195	NC_030826.1
novel_circ_0003560	circ_LOC106502060	up	--	0.006314	exon	25358995	25385681	+	252	NC_030811.1
novel_circ_0012647	circ_TAGLN	up	--	0.033282	exon	54452454	54453324	-	473	NC_030822.1
