# Supplementary material for: Integrating Analysis to Identify Differential circRNAs Involved in Goat Endometrial Receptivity
Source: Int J Mol Sci. 2023 Jan 12;24(2):1531. doi: 10.3390/ijms24021531 (PMC9865150; doi:10.3390/ijms24021531)
Supplement: Supplementary file 1 [file ijms-24-01531-s001.zip › Supplementary Table S7.pdf]

Supplementary Table S7. Primers used in the present study.

Name	Sequence (5′- 3′)
circ_ZNF568-F	AGAACTACAGCAACCTAGTTACAG
circ_ZNF568-R	TGCTCTTTATAAGAGACAGACCTG
ZNF568-F	ATTGCTAGACCTGCACCCTG
ZNF568-R	ATGCCAAGCTGTCCTTTCCA
circ_LOC106502060-F	AAGATGAGGAGTTAAGAGCT
circ_LOC106502060-R	CCAAACTCTCTTTACCAGTT
LOC106502060-F	GGACCTACTTTGTGGGAGCAA
LOC106502060-R	TGCTGCATAAATGGTTGCCT
circ_CRIM1-F	CAGCTACGAAACGCAAGTCA
circ_CRIM1-R	AACGTGGAGAGAACTGGACT
CRIM1-F	TCCAGTTCTCTCCACGTTGTC
CRIM1-R	TATCCTGGCTGGCAGACTTTC
circ_ MYRF-F	TCTGCAGGGTCAATCTGCCC
circ_ MYRF-R	TGCTTCGGTCCGACTGCGAC
MYRF-F	CAGTCAGACCCAGTGCCATT
MYRF-R	GCTGCTAACAGGGATGTGGT
circ_LOC106502447-F	TGACAGCTGGGCAGATGTTC
circ_LOC106502447-R	TCCACTGATGTGCTCTTCCA
novel_circ_0007697-F	AGGTCAGGCTCATCACATCT
novel_circ_0007697-R	ACGTCCATCACATTGCATCA
ISG15-F	AACCTGACACCAGAACCCAC
ISG15-R	TTCTGGGCGATGAACTGCTT
WNK1-F	TCCCGTTCCCACAATGTCTG
WNK1-R	CCGAGCTGGAGCCATTCTT
GAPDH-F	TGCCCGTTCGACAGATAGC
GAPDH-R	ACGATGTCCACTTTGCCAGTA
